# Maintaining Myofibroblastic-Like Cancer-Associated Fibroblasts by Cancer Stemness Signal Transduction Feedback Loop

**DOI:** 10.7759/cureus.29354

**Published:** 2022-09-20

**Authors:** Michael P Rogers, Anai Kothari, Meagan Read, Paul C Kuo, Zhiyong Mi

**Affiliations:** 1 Department of Surgery, University of South Florida Morsani College of Medicine, Tampa, USA; 2 Department of Surgery, Medical College of Wisconsin, Milwaukee, USA

**Keywords:** feedback loop, cancer associated fibroblast, mda-mb-231, tumor microenvironment, cancer stemness

## Abstract

Background: Myofibroblast-like cancer-associated fibroblasts (myCAF) in the tumor microenvironment (TME) promote cancer stemness, growth, and metastasis. Cancer cell-derived osteopontin (OPN) has been reported as a biomarker related to malignant cancer growth. In this study, we confirm that cancer cell stemness is required for the maintenance of an OPN-induced myCAF phenotype. Methods: MDA-MB-231 or HepG2 cells and Sox2 knockout variants were co-cultured with human mesenchymal stem cells (MSC). In selected instances, the OPN bioactivity inhibitor OPN-R3 aptamer (APT), OPN-R3 mutant aptamer (MuAPT), or cancer cell stemness inhibitor BBI-608 were added separately. MDA-MB-231 cancer stemness and myCAF markers were quantified by real-time PCR. Stemness-lacking cancer cell mice models were created to confirm that stemness is required for the maintenance of the OPN-induced myCAF phenotype *in vivo*. Results: In an MDA-MB-231 co-culture system, myCAF and stemness markers increased. Osteopontin and stemness blockade in this co-culture system decreased both myCAF and stemness marker expression, but OPN blockade after 72 hours had no effect. In contrast, when BBI608 was added at 72 hours, myCAF markers were abated after 36-hour treatment. Replacing wildtype with MDA-MB-231(-/-sox2) in co-cultures at 72 hours decreased myCAF marker expression to baseline despite the Western blot confirming the presence of OPN. Conversely, replacing MDA-MB-231(-/-sox2) cells with wildtype increased myCAF marker expression to a level equivalent to the MDA-MB-231+MSC co-culture system. *In vivo* osteopontin blockade diminished stemness and myCAF marker expression and stemness lacking cancer cell models, indicated by decreasing myCAF presence. Experiments were repeated in a HepG2 cell line with identical results. Conclusions: Cancer and myCAF crosstalk increases myCAF maintenance and cancer cell stemness. In this study using human breast and liver cancer cell lines, maintenance of the OPN-induced myCAF phenotype also requires cancer stemness. This indicates that the myCAF phenotype requires two distinct signaling pathways: initiation and maintenance.

## Introduction

The understanding of carcinogenesis at the cellular, genetic, molecular, and microenvironmental levels continues to progress. The focus of intense research has been on elucidating factors that influence cancer cell regulation, invasion, growth, and metastatic potential. Cumulative evidence has revealed components of the tumor microenvironment (TME), including the extracellular matrix, fibroblasts, myofibroblasts, macrophages, lymphocytes, mesenchymal cells, and myofibroblast-like cancer-associated fibroblasts (myCAF), interact through complex networks of cytokines, growth factors, and mitogens to promote tumor growth [1,2]. Further, myCAF within the TME has been implicated in promoting cancer stemness, tumor growth, and metastasis [3-7]. Consequently, recent attention has been directed at targeting myCAF numbers or functions to potentially impact cancer therapeutic offerings [8-10].

Cancer cell-derived osteopontin (OPN), a glyco-phosphoprotein involved in cell adhesion, chemotaxis, macrophage-directed interleukin-10 suppression, and prevention of cellular apoptosis, is a marker for aggressive cancer growth [11,12]. Tumor-derived OPN has been shown to prompt bone marrow-derived mesenchymal stem cell (MSC) trafficking to the TME [13,14]. Through OPN-induced myCAF transformation and tumor microenvironment crosstalk, cancer stemness marker expression (Oct4, Nanog, and Sox2) and human breast cancer metastasis are highly increased [15]. Additionally, Sox2 knockdown has been shown to result in decreased cancer stemness [16-19]. Our lab’s previous work indicates the expression of OPN secreted from high-metastatic breast cancer cells is much higher than the low-metastatic breast cancer cells either in human or mouse models [14,20,21].

Previous work has demonstrated the OPN-induced initiation of mesenchymal stem cell adoption of the myCAF phenotype with a consequent juxtacrine increase in MDA-MB-231 cell and HepG2 cell stemness, using separate co-culture models of human MDA-MB-231 breast cancer and Hep-G2 liver cancer [13,22-24]. However, while the influence of myCAF on cancer cell stemness has been extensively characterized, little work has been performed examining the influence of stemness on myCAF. Here, we demonstrate cancer cell stemness is required for maintenance of the OPN-induced myCAF phenotype-culture conditions.

## Materials and methods

Materials

The synthesis of OPN-R3 aptamer (APT) and OPN-R3 mutant aptamer (MuAPT) was described previously [22]. The OPN Ab (sc-21742), Sox2 Ab (sc-17320), and Sox-2 shRNA lentiviral particles (sc-38408-v) were purchased from Santa Cruz Biotechnology, Santa Cruz, CA. The β-actin Ab (4967) was purchased from Cell Signaling Technology, Danvers, MA. The Napabucasin (BBI608, 5.33851) was purchased from Sigma-Aldrich, St. Louis, MO.

Cell culture

Human MSCs were kindly provided by the Texas A&M Institute for Regenerative Medicine (Bryan, TX) and maintained by following their instructions [25]. The human MDA-MB-231 and Hep-G2 cell lines were purchased from the American Type Culture Collection, respectively. Transfection with Sox-2 shRNA lentiviral particles to generate MDA-MB-231 and Hep-G2 cell lines with constitutive knockdown of Sox-2 was followed by the company’s protocol.

Co-culture

The co-culture experiments were performed with Boyden Chamber plates (Corning, Inc., Corning, NY) by following the instructions in their product manual. Briefly, the human MSC cells and the cancer cells were seeded in the upper and lower chambers, respectively, or vice versa. The upper chamber and the lower chamber were divided by the 0.4 µm pore size wells, which physically separate these two different cells but allow them to perform paracrine signaling.

Western blot

Previously, Western blot procedures were reported [26]. The primary antibody dilutions are 1:1000 for OPN Ab, 1:1000 for Sox2 Ab, and 1:1000 for β-Actin Ab.

Mouse xenograft model

All animal experimental procedures were conducted by following the National Institutes of Health guidelines and approved by the IACUC committee of Loyola University of Chicago (IACUC#203984), which is certified by the Association for Assessment and Accreditation of Laboratory Animal Care (AAALAC). The detailed procedures were reported previously [13]. Briefly, six-week-old female immunocompromised NOD scid gamma mice (strain#005557) were received from the Jackson Laboratory (Bar Harbor, Maine). The 106 MDA-MB-231 cells or SK-Hep1 sox2 shRNA knock down-RFP-luciferase-expressing cells and/or 106 GFP-labeled human MSC cells were diluted with 50 µl of PBS solution and implanted into the mammary fat pad at the R4 site. 10 mg/kg of OPN-R3, or OPN-R3mut, was applied for the treatments through tail vein injection. These treatments were performed every two days, starting from the cancer cells' implantation time point, and continued for eight weeks. The Perkin Xenogen IVIS-100 system was used to quantify the bioluminescence signals of the anesthetized mice every week by following its manual. Shortly, the anesthetized mice were placed inside the imaging chamber after 10 minutes of the D-luciferin intraperitoneal injection (150 mg/kg). The bioluminescence signals are quantified by the Living Image Software (Perkin Elmer, Houston, Texas).

Fluorescence activated cell sorting

Single-cell suspensions were prepared with the collection of primary tumors, lungs, or liver tissues as we reported previously [13]. Briefly, the tissues were finely minced and transferred to the tubes containing 10 ml dissociation medium (0.025% collagenase, 0.05% pronase, and 0.04% DNase in 1 × PBS, PH 7.4). After the tubes were incubated at 37 °C for one hour, they were centrifuged for 10 min (300 RCF, at 4 °C) and the tissue cell pellets were gently washed three times with PBS solution (5 ml each time, at 4 °C). The homogenate tissue suspension (1 × PBS, PH 7.4, at 4 °C) was gently filtered through a nylon mesh filter (70 µm pore size, at 4 °C). Cells were sorted using BD FACSAriaIII (BD Biosciences, Franklin Lakes, NJ). The Aldefluor kit (Stemcell Technologies, Cambridge, MA) was applied for aldh positive cell sorting following its manual. For GFP-positive cell sorting, the laser excitation is 488 nm (band-pass filter 530/30). For RFP positive cell sorting, the laser excitation is 561 nm (band-pass filter 610/20). The sorted cells were collected in PBS and stored at −80 °C until further analysis.

Immunohistochemistry

The immunohistochemistry staining and imaging were performed by the Research Histology and Tissue Imaging Core at the University of Illinois Chicago.

Quantitative real-time PCR

The procedures for total RNA isolation (TRIzol, Invitrogen) and cDNA synthesis (iScript, Bio-Rad Laboratories) were performed by following the manufacturer's manuals. The quantitative real-time PCR protocols and the primer sequences for detecting α-smooth muscle actin (α-SMA)/vimentin (VIM)/tenascin-C (TEN) gene expression were reported previously [15]. The ΔΔCT values were calculated after the b-actin normalization.

Primer Sequences

Sox2: 5′-GCCTGGGCGCCGAGTGGA-3′; 5′-GGGCGAGCCGTTCATGTAGGTCTG-3′ (length: 443bp)

Oct4: 5′-GCTCGAGAAGGATGTGGTCC-3′; 5′-CGTTGTGCATAGTCGCTGCT-3′ (length: 81bp)

Nanog: 5′-TCTGGACACTGGCTGAATCCT-3′; 5′-CGCTGATTAGGCTCCAACCAT-3′ (length: 144bp)

b-actin: 5′-AGCGGGAAATCGTGCGTGAC-3′; 5′-CAATGGTGATGACCTGGCCGT-3′ (length: 135bp)

myCAF cell imaging

The myCAF co-culture images were taken at 24 hours and 48 hours, respectively, by using a Leica SP2 confocal microscope.

Cancer cells migration assays

The migration assays were performed with Boyden Chamber plates following the manufacturer's manual. Mesenchymal stem cells were seeded at 102 cells per well in the lower chamber of 12 trans-well plates (8 µm pore). 1 × 10^3^ cells per well of MDA-MB-231 cells, HepG2 cells, or their Sox2 knockdown cells were seeded in the upper chambers. 100 nM OPN-R3 or OPN-R3mut control were added to the co-culture system and proceeded for 48 hours. The trans-well membranes were fixed in paraformaldehyde solution (3.7% in 1 × PBS) for 10 min. The cells attaching to the upper surface of the membranes were wiped out with swabs. After three washes with 1 × PBS, the filters were stained with crystal violet (0.4%) for 10 minutes. After extensive rinsing to remove the non-stable stains, the images were taken with an Olympus I×73 inverted microscope.

Statistical analysis

All experiments were done in triplicate. Data are presented as mean ± standard deviation. Student’s t-tests and ANOVA were used for analysis where appropriate. Values of p<0.05 were considered statistically significant.

## Results

Cancer-secreted OPN induces MSC adoption of the myCAF phenotype resulting in increased cancer stemness in MDA-MB-231 cells and HepG2 cells

**Figure 1:**
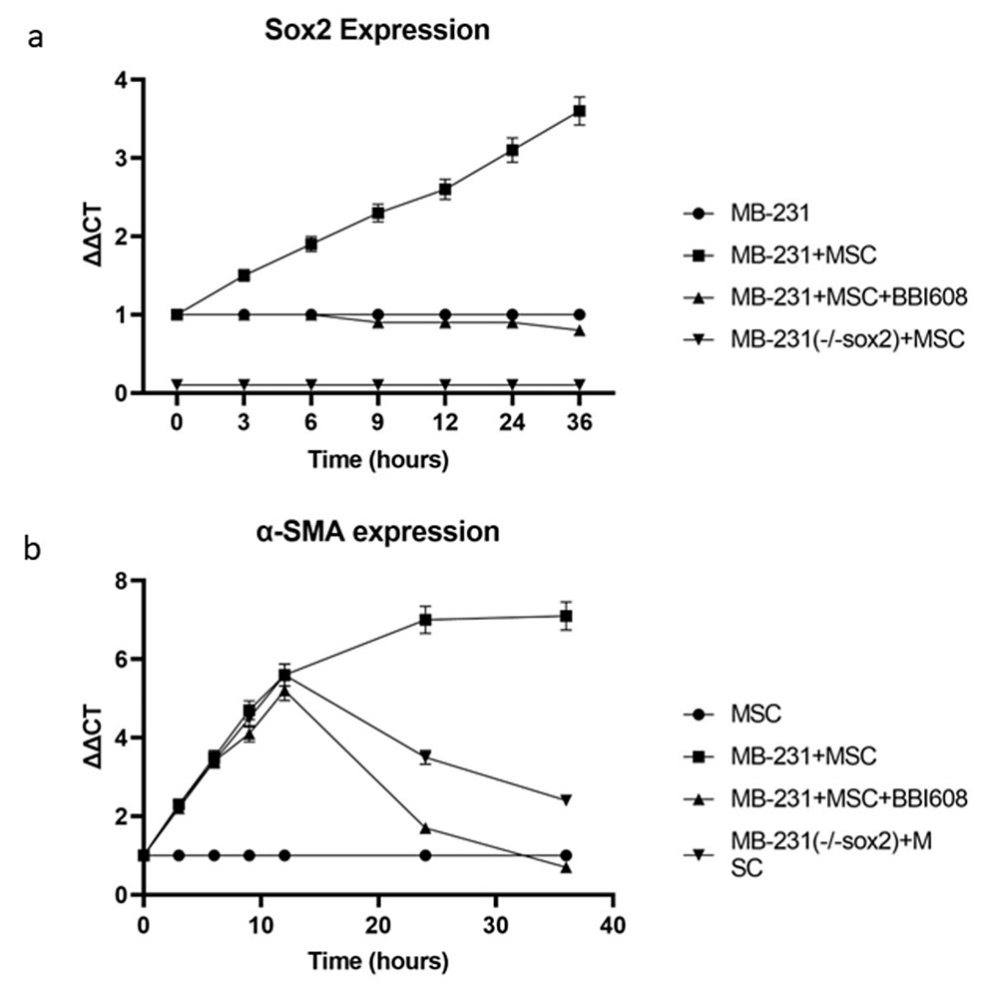
Cancer secreted OPN induces MSC adoption of myCAF phenotype resulting in increased cancer stemness Co-culture of wild type MDA-MB-231 cells with human MSC cells and cancer stemness lacking MDA-MB-231(-/-sox2) cells with human MSC cells. Cancer stemness inhibitor BBI-608 was added as indicated: (A) cancer stemness marker Sox2 mRNA expression was quantified by real time PCR and (B) myCAF marker α-SMA mRNA expression was quantified by real time PCR.

In a model using the MDA-MB-231 human breast cancer and HepG2 human hepatocellular carcinoma cell lines co-cultured with human MSCs (data shown in supplemental), the well-established myCAF markers including α-SMA, VIM, and TEN, increased approximately sevenfold and plateaued at 48-hours (Figure 1, MDA-MB-231 VIM/Ten-C shown in supplemental, Hep-G2 data not shown).

In our previous work in this model, we demonstrated cancer-secreted OPN acts through the transcription factor MZF1 to express the myCAF phenotype corresponding with increased α-SMA, VIM, and TEN-C [13]. Additionally, the cancer stemness markers Oct-4, Nanog, and Sox2 increased approximately fivefold in each cell line (Figure 1, Nanog and Oct-4 data shown in supplemental). These results are consistent with previous work demonstrating that MDA-MB-231-derived OPN acts on human MSCs in co-culture to express a myCAF phenotype with concomitant feedback to MDA-MB-231 to increase cancer stemness [15].

**Figure 2:**
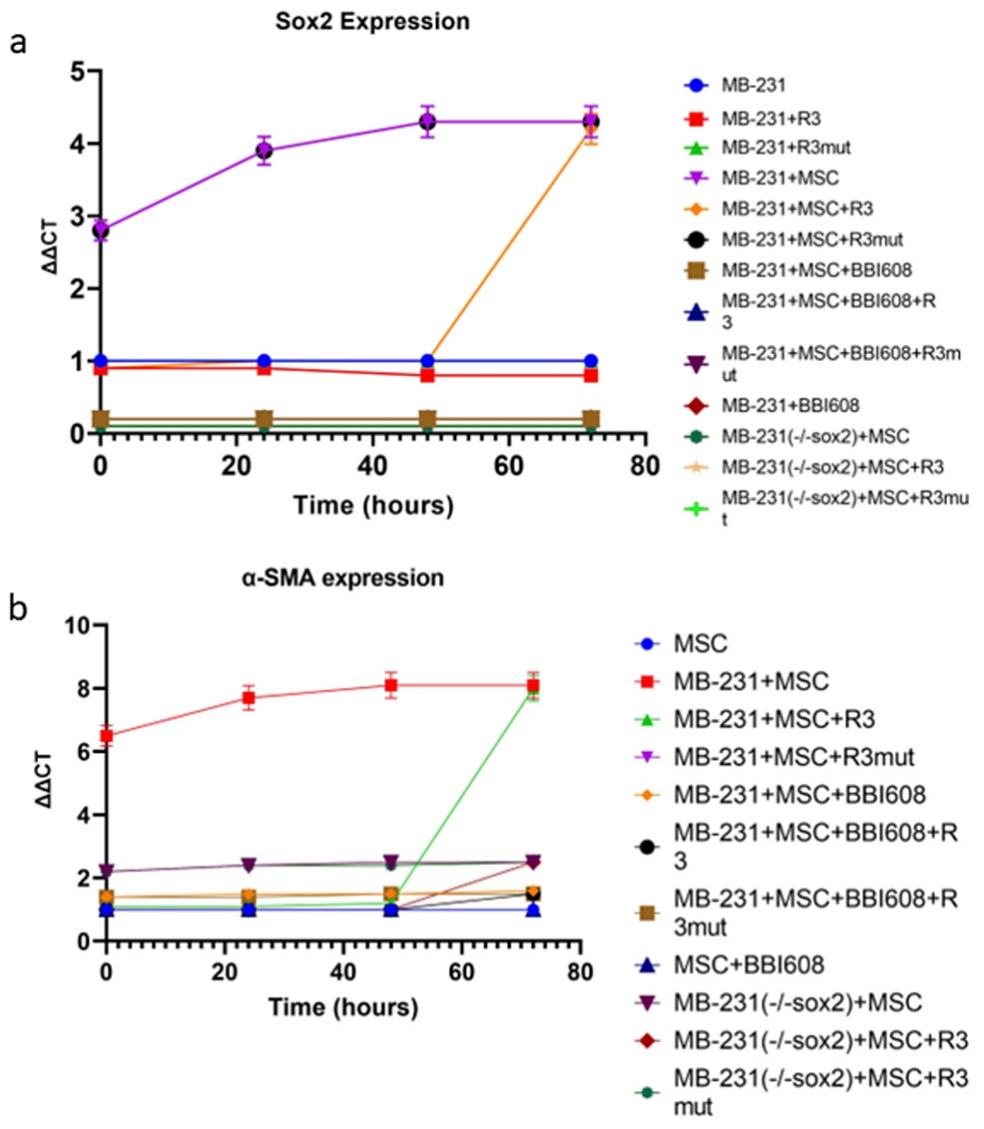
Cancer stemness is required to maintain the myCAF phenotype MDA-MB-231 cells or cancer stemness lacking MDA-MB-231(-/-sox2) cells were co-cultured with human MSC cells. OPN bioactivity inhibitor OPN-R3 or cancer stemness inhibitor BBI608 were added, respectively, at the indicated time points and kept for 24 hours. (A) Cancer stemness marker Sox2 mRNA expression was quantified by real time PCR and (B) myCAF marker α-SMA mRNA expression was quantified by real-time PCR.

Osteopontin blockade with the RNA aptamer OPN-R3 (APT) at zero hours in both cell lines ablated the increase in the myCAF phenotype and cancer stemness markers (p<0.05 vs OPN, Figure 2).

Results for MDA-MB-231 and HepG2 levels of other stemness and myCAF markers were similar (data not shown). However, the OPN blockade for 72 hours had no effect.

Cancer stemness in MDA-MB-231 and HepG2 is required to maintain the myCAF phenotype

**Figure 3:**
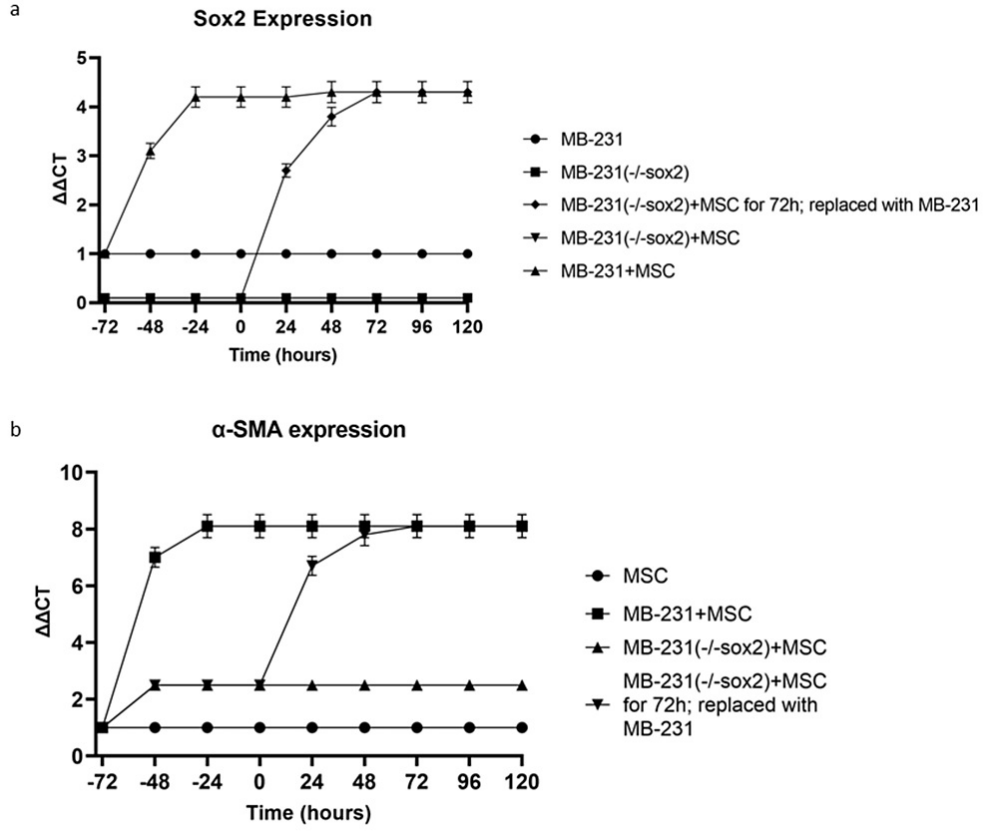
Cancer stemness is required to maintain the myCAF phenotype MDA-MB-231 cells or cancer stemness lacking MDA-MB-231 (-/-sox2) cells were co-cultured with human MSC cells for 72 hours. After 72 hours co-culture, MDA-MB-231 (-/-sox2) cells were replaced with MDA-MB-231 cells. At the indicated time points, cells were harvested. (A) Cancer stemness marker Sox2 mRNA expression was quantified by real time PCR and (B) myCAF marker α-SMA mRNA expression was quantified by real-time PCR.

In an MDA-MB-231+MSC (or HepG2+MSC) co-culture, MDA-MB-231(-/-sox2)+MSC was replaced with MDA-MB-231(sox2) after 72 hours, yielding myCAF marker expression commensurate with wild-type MDA-MB-231+MSC at 72 hours (and HepG2+MSC, Figure 3).

Co-culture studies were repeated for other markers of cancer stemness (Oct4, Nanog, data in supplemental). Expression of the OPN-mediated myCAF markers (α-SMA, VIM, TEN-C) was similarly evaluated with the replacement of MDA-MB-231(-/-sox2) with wild-type MDA-MB-231+MSC after 72 hours, demonstrating myCAF marker expression equivalently increasing to wild-type levels.

Real-time PCR of MDA-MB-231 with increasing concentrations of a small molecular cancer stemness inhibitor, BBI-608, demonstrated stepwise decreases in expressions of Nanog, Sox2, and Oct4 stemness markers (shown in supplemental data). The addition of BBI-608 impeded myCAF marker expression in MDA-MB-231+MSC and HepG2+MSC cell lines (p < 0.05 vs MDA-MB-231 (or HepG2)+MSC).

Cancer secreted OPN initiates myCAF but does not maintain the myCAF phenotype

**Figure 4:**
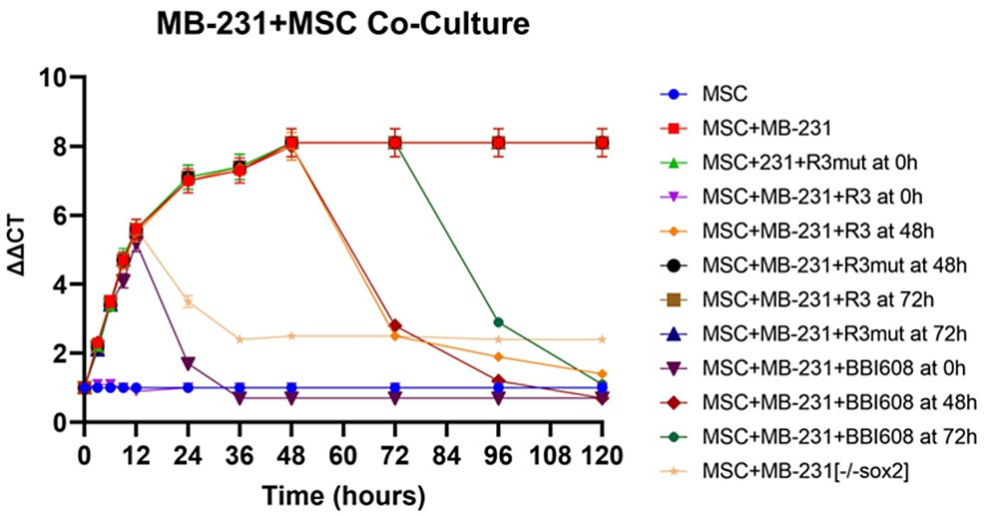
Cancer stemness is required to maintain the myCAF phenotype MDA-MB-231 cells were co-cultured with MSC cells for 120 hours. OPN bioactivity inhibitor OPN-R3 or cancer stemness inhibitor BBI608 was added at the co-culture time points 0 h, 48 h, and 72h, respectively. Cells were harvested at the indicated time points, myCAF marker α-SMA mRNA expression was quantified by real time PCR.

Myofibroblastic like cancer-associated fibroblast markers' expression reliance on OPN was evaluated in MDA-MB-231+MSC (or HepG2+MSC) with OPN or myCAF blockade at various time points using APT and BBI-608, respectively (Figure 4).

Osteopontin blockade with APT after 48-hour or 72-hour co-culture demonstrated corresponding decreases in α-SMA expression. However, MDA-MB-231+MSC+MuAPT continued to express α-SMA at a level comparable with wild-type MDA-MB-231+MSC. MDA-MB-231+MSC (-/-sox2) co-culture initially expressed α-SMA levels comparably with the wild type; however, it peaked at 12 hours and subsequently decreased. Similarly, BBI-608 resulted in decreased α-SMA expression when introduced at 48-hour and 72-hour intervals. However, MDA-MB-231+MSC (or HepG2+MSC) with BBI-608 at 0 hours showed an initial increase in α-SMA expression, with a subsequent fall in expression after six hours of co-culture.

**Figure 5:**
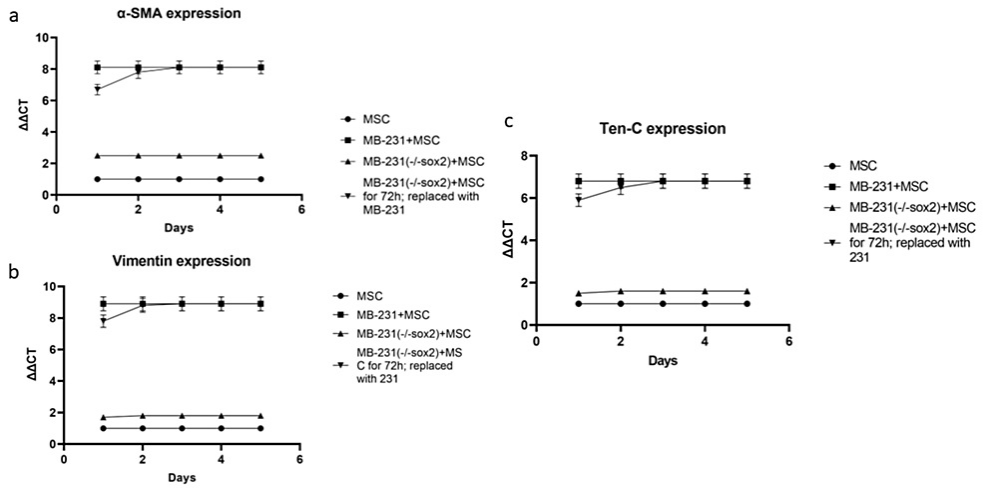
Cancer stemness is required to maintain the myCAF phenotype MDA-MB-231 cells or cancer stemness lacking MDA-MB-231(-/-sox2) cells were co-cultured with human MSC cells for 72 hours. After 72 hours co-culture, MDA-MB-231(-/-sox2) cells were replaced with MDA-MB-231 cells. At the indicated time points, cells were harvested (A-C) myCAF markers α-SMA/Vim/Ten-C mRNA expression was quantified by real time.

After a 72-hour co-culture of wild-type MDA-MB-231+MSC (or HepG2+MSC), MDA-MB-231(-/-sox2), or HepG2(-/-sox) replaced wild-type cells with a resultant decrease in myCAF markers expression to baseline levels of MDA-MB-231+MSC(-/-sox2), despite Western blot confirmation of OPN presence with Sox-2 shRNA lentivirus knocking down. Conversely, the replacement of MDA-MB-231(-/-sox2) (or HepG2 [-/-sox2]) with wild-type MDA-MB-231 (or HepG2[-/-sox2] with wild-type HepG2) increased myCAF markers to an equivalent level to MDA-MB231+MSC alone (p < 0.05, Figure 5).

In vivo osteopontin blockade diminishes cancer stemness and myCAF marker expression in stemness lacking cancer cell models indicated by decreasing myCAF presenting in the TMEN

**Figure 6:**
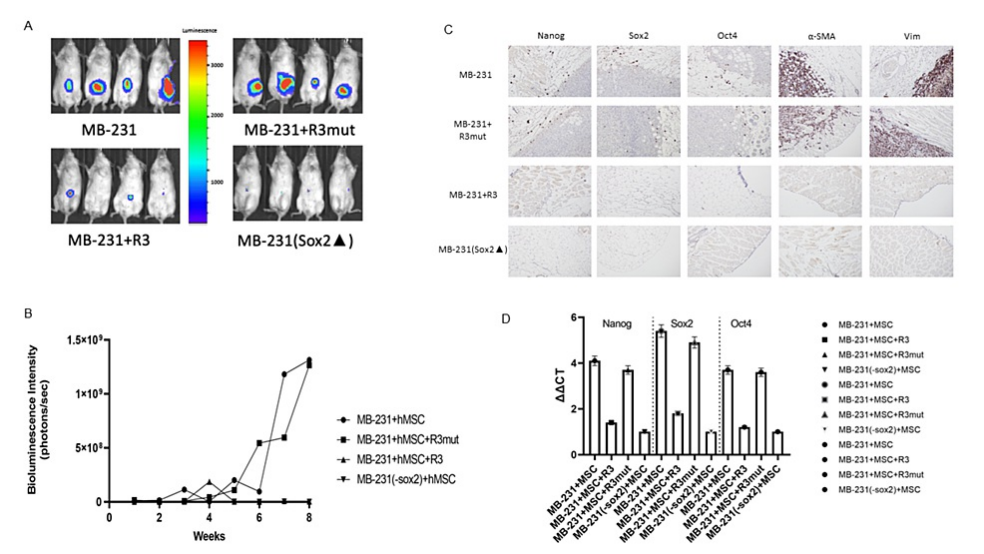
Cancer stemness is required to maintain the myCAF phenotype in cancer mice models (A) In vivo imaging of bioluminescence labeled MDA-MB-231 cells breast cancer mice models at week 8; (B) bioluminescence intensity-based tumor growth curves; (C) immunohistochemistry assay of cancer stemness markers expression and myCAF markers expression in mice primary tumors; (D) cancer stemness markers mRNA expression and myCAF markers mRNA expression were quantified by real time PCR with mice primary tumor sorted cells.

In a murine model, MDA-MB-231 and SK-Hep1 (wild-type or stemness lacking) cell lines (SK-Hep 1 data showed in supplement) were tagged with luciferase and RFP and co-injected with GFP-tagged MSC to the immunocompromised mice, OPN-R3 (APT), MuAPT (R3Mut) were applied for the treatments. Luciferase activity was quantified weekly through IVIS imaging. In control cell lines (MDA-MB-231 and SK-Hep1), tumor formation was noted at approximately three weeks and significantly increased by week 6. Tumor formation and bioluminescent intensity in the MuAPT cohort paralleled MDA-MB-231 control, reaching an intensity of 1.3 × 10^9^ photons/s/cm^2^/sr at eight weeks (Figure 6A).

Bioluminescence intensity increased in the SK-Hep1 cell line, however the MuAPT cohort did not reach the same intensity as control (2.1 × 10^9^ vs 7.9 × 10^8^ photons/s/cm^2^/sr). The APT cohort and (-/-)sox2 cohorts did not experience significant changes in luminescence or tumor growth over eight weeks (Figure 6B). 

Following eight weeks of tumor growth, the murine models were sacrificed, and immunohistochemistry performed on the tumor (Figure 6C). MDA-MB-231 and SK-Hep1 controls and OPN-R3 groups demonstrated intense expression of stemness markers along the periphery of the tumor. The MuAPT and (-/-sox2) cohorts demonstrated a decrease in tumor cell and stemness marker staining. Real time PCR results demonstrated significant decreases in Nanog, Sox2, and Oct4 expression in MuAPT and (-/-sox2) cohorts (Figure 6D). These results paralleled with our in vitro experiment results confirmed that OPN blockade diminishes cancer stemness and myCAF markers expression. Stemness lacking cancer cells models indicated decreasing myCAF presenting in the TME.

## Discussion

**Figure 7:**
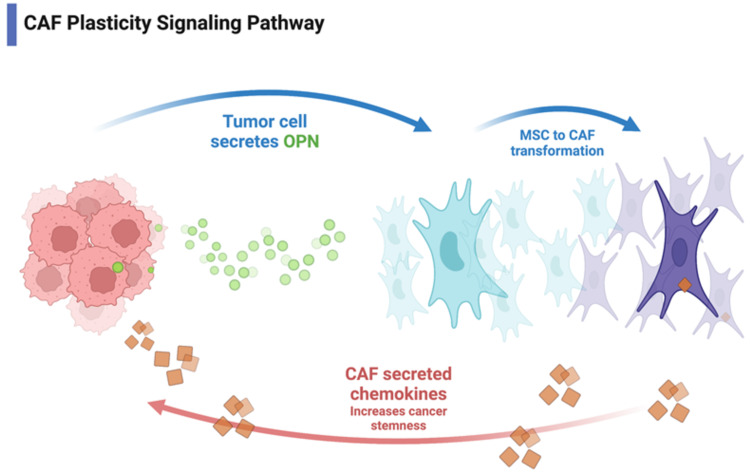
Myofibroblast-like CAF plasticity pathway Tumor cells secrete osteopontin which induces MSC to myCAF transformation. myCAF phenotype increases cancer stemness through secreted chemokines (the image made by our lab).

In this study, we demonstrate the requirement of cancer cell stemness for maintenance of the osteopontin-induced myCAF phenotype in MBA-MB-231 breast cancer and HepG2 hepatocellular carcinoma cell lines through cancer cell-myCAF crosstalk. Additionally, the osteopontin-induced myCAF phenotype requires cancer stemness for myCAF maintenance and potentiation. Blockade of cancer stemness using the small molecular stemness inhibitor BBI-608 results in decreased myCAF marker expression. These findings indicate that the myCAF phenotype requires two distinct signaling pathways: initiation and maintenance (Figure 7).

Tumor heterogeneity is thought to be dependent on distinct cancer stem cells (CSCs), which have the ability to sustain tumor growth, self-renew, and differentiate into multiple cell types [27]. CSCs and myCAFs are thought to interact in the TME and support each other through reciprocal signaling [6,28,29]. Previous work by Valenti et al. has confirmed myCAFs role in proliferating and maintaining CSCs in mouse mammary epithelial cells [6]. Further, myCAFs have been implicated in altering the invasive nature of breast cancer CSCs and may promote the transition of tumors to invasive phenotypes [6]. Herein, we demonstrate that cancer cell stemness, as measured by increased expression of the pluripotency-associated transcription factors Sox2, Nanog, and Oct4, appears to be required for the maintenance and potentiation of the myCAF phenotype. This maintenance may influence myCAF’s ability to contribute to a tumor-permissive inflammatory environment and the display of tumor-promoting and pro-metastatic properties, including the production of angiogenic factors and matrix metalloproteinases [30].

## Conclusions

Cancer and myCAF cell crosstalk increase myCAF cell maintenance and cancer cell stemness. In this study using human breast and liver cancer cell lines and cancer mice models, we discovered that the myCAF phenotype requires two distinct and parallel signaling pathways, initiation and maintenance. This is a unique and novel finding that provides a rationale for further investigation into elucidating these mechanisms.

## Figures and Tables

**Figure 8:**
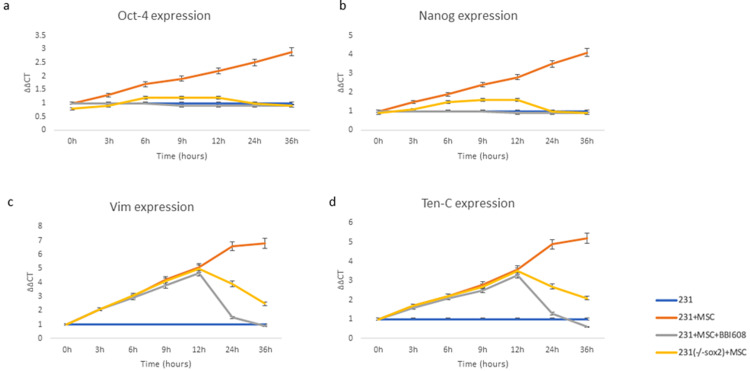
Cancer-secreted OPN induces MSC adoption of myCAF phenotype resulting in increased cancer stemness Co-culture of wild type MDA-MB-231 cells with human MSC cells and cancer stemness lacking MDA-MB-231(-/-sox2) cells with human MSC cells. Cancer stemness inhibitor BBI-608 was added as indicated (A,B) Cancer stemness markers Oct4/Nanog mRNA expression were quantified by real time PCR. (C,D) myCAF markers Vim/Ten-C mRNA expression were quantified by real time PCR.

**Figure 9:**
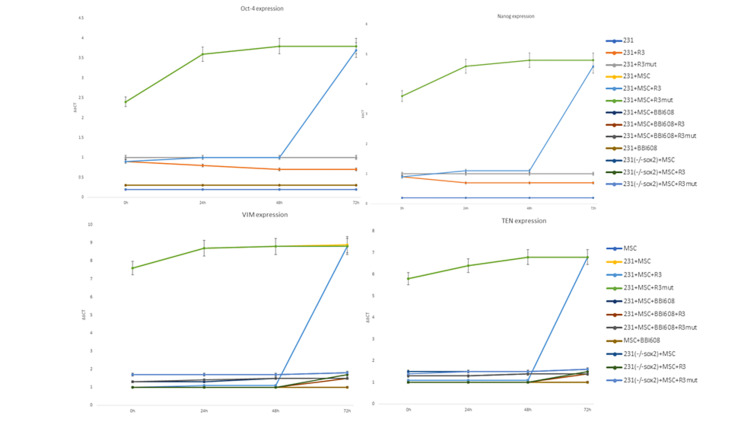
MB-231+MSC Oct4, Nanog, VIM, and Ten-C expression MB-231+MSC with APT (R3), APT-Mutant, BBI-608, and MB-231(-/-sox) co-culture on Oct4, Nanog, VIM, and Ten-C expression by real time PCR.

**Figure 10:**
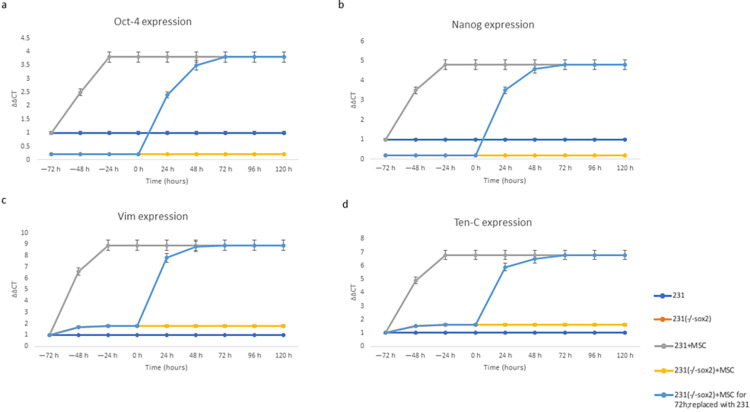
Cancer stemness is required to maintain the myCAF phenotype MDA-MB-231 cells or cancer stemness lacking MDA-MB-231(-/-sox2) cells were co-cultured with human MSC cells for 72 hours. After 72 hours co-culture, MDA-MB-231(-/-sox2) cells were replaced with MDA-MB-231 cells. At the indicated time points, cells were harvested. (A,B) Cancer stemness markers Oct4/Nanog mRNA expression were quantified by real time PCR. (C,D) myCAF markers Vim/Ten-C mRNA expression was quantified by real time PCR.

**Figure 11:**
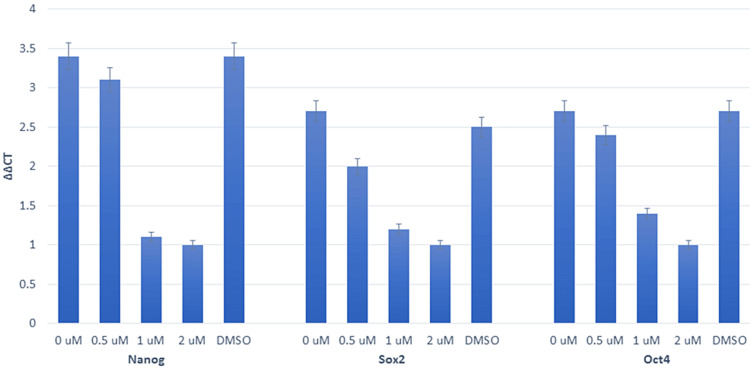
Cancer stemness is required to maintain the myCAF phenotype MDA-MB-231 cells were treated with cancer stemness inhibitor BBI608 at different concentrations for 48 hours. Cancer stemness markers Sox2/Oct4/Nanog mRNA expression were quantified by real time PCR.

**Figure 12:**
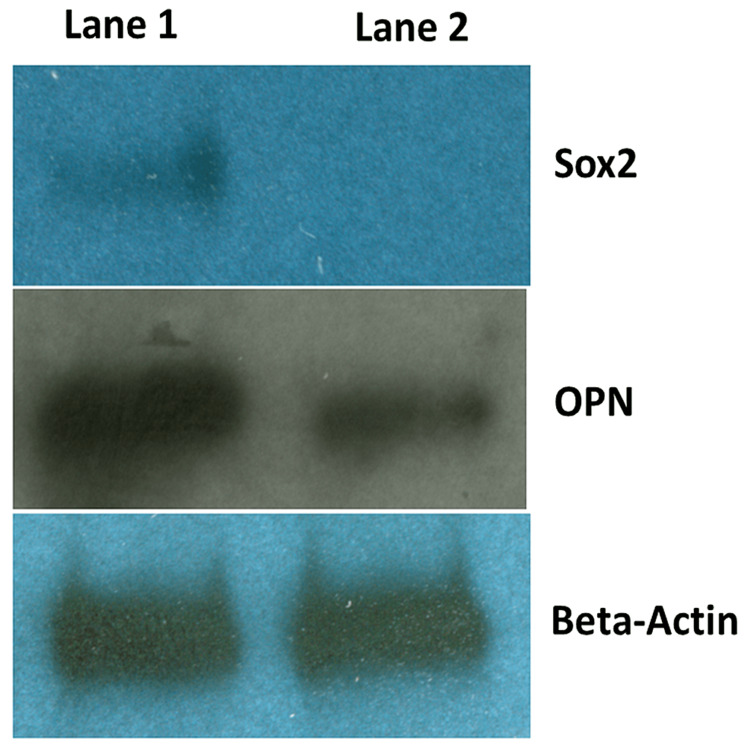
shRNA lentivirus knocking down sox2 expression in MDA-MB-231 cells Western blot was performed to quantify sox2, OPN protein expression in (Lane #1) MDA-MB-231 cells and (Lane #2) sox2 shRNA lentivirus stable transfected MDA-MB-231(-/-sox2) cells. Βeta-actin was chosen as house-keeping gene expression control.

**Figure 13:**
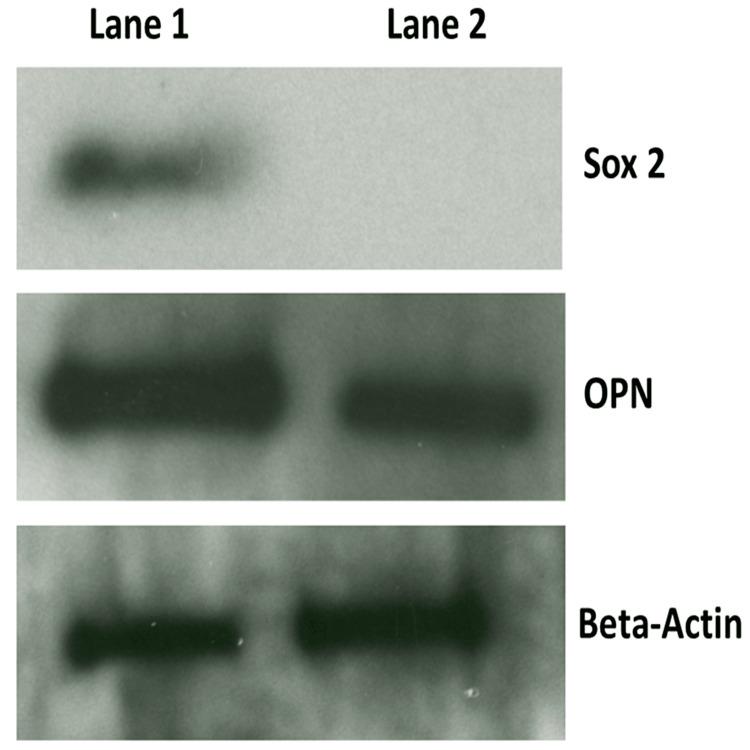
shRNA lentivirus knocking down sox2 expression in Hep-G2 cells Western blot demonstration of Sox2, OPN, and beta-actin presence in Hep-G2 (lane #1) compared with Hep-G2+Sox2 shRNA lentivirus (lane #2). Βeta-actin was chosen as house-keeping gene expression control.

**Figure 14:**
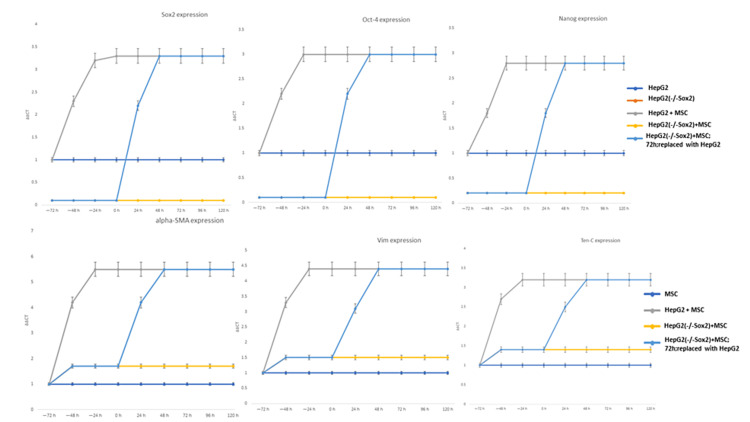
Hep-G2+MSC co-culture expression of stemness and CAF markers Hep-G2+MSC co-culture, Hep-G2(-/-sox2)+MSC was replaced with Hep-G2(sox2) after 72 hours, yielding expression equivalent with wildtype Hep-G2+MSC at 72 hours by real time PCR.

**Figure 15:**
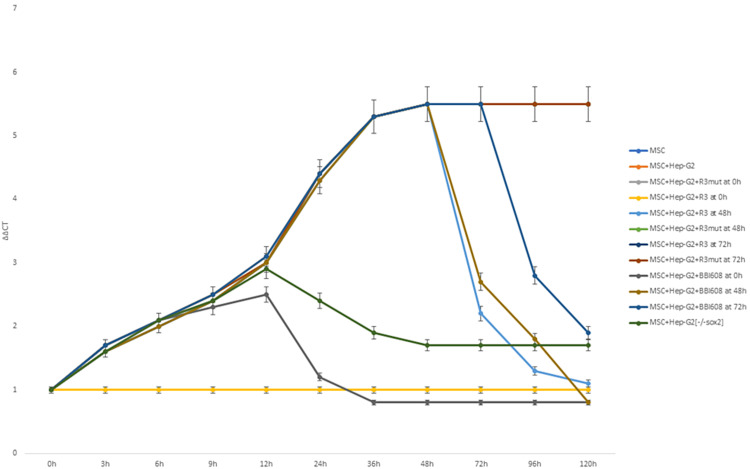
SMA expression in Hep-G2+MSC co-culture SMA expression in Hep-G2+MSC co-culture with APT (R3), APT-MUT (MuAPT), BBI-608, and Hep-G2+MSC(-/-sox2).

**Figure 16:**
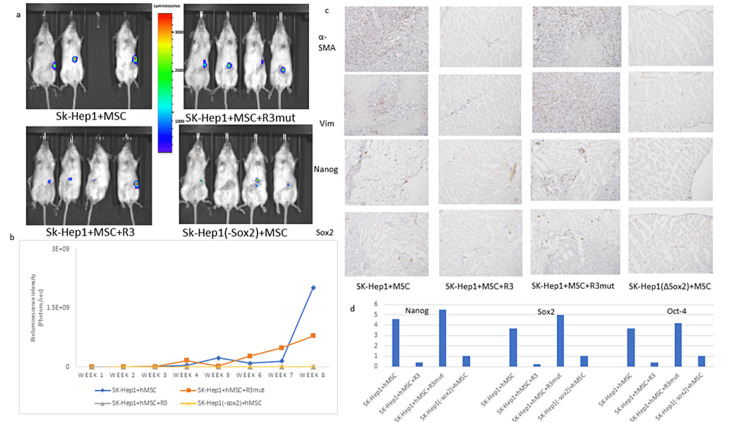
Cancer stemness is required to maintain the myCAF phenotype in murine cancer models (A) In vivo imaging of bioluminescence labeled SK-Hep1 human liver cancer murine models at week; (B) bioluminescence intensity-based tumor growth curves; (C) immunohistochemistry assay of cancer stemness markers expression and myCAF markers expression in mice primary tumors; (D) cancer stemness markers mRNA expression and myCAF markers mRNA expression were quantified by real time PCR with mice primary tumor sorted cells.

**Figure 17:**
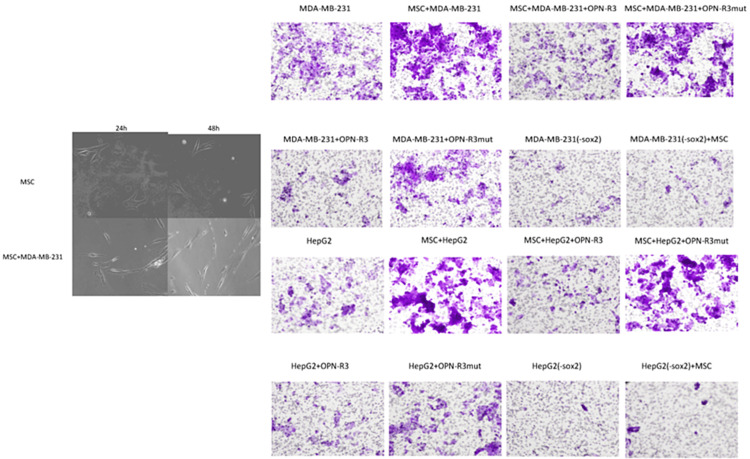
myCAF morphology imaging and myCAF migration assays Figure 10 (supplemental powerpoint slides 18-19) myCAF morphology imaging and myCAF migration assays in MBA-MB-231 and HepG2 cell lines under indicated co-culture conditions.

## Appendices

Supplemental data
